# Spiritus Pyroxylicus in the Treatment of Diarrhea and Dysentery

**Published:** 1849-09

**Authors:** David W. Yandell

**Affiliations:** Louisville, Ky.


### WESTERN JOURNAL

OF

### MEDICINE AND SURGERY.

SEPTEMBER, 1849.

### Art. I.—*Spiritus Pyroxylicus in the Treatment of Diarrhea and Dysentery.* By David W. Yandell, M. D., of Louisville, Ky.

My attention was first called to the use of pyroxylic spirit, or medicinal naptha, in diarrhea, by an article which I translated for the June number of this Journal, contained in the *Gazette des Hopitaux.* There, the efficacy of this agent was so confidently affirmed by Dr. Lavirotte, of Lyons, who, I believe, was the first to use it in this disease at least, in France, that I myself was induced to make trial of it in diarrhea, and by analogy was subsequently led to administer it also in dysentery. The result of this trial I now purpose making known to the profession.

*Case* 1. *Diarrhea.*—Mr. Johnson, a native of the West Indies, æt. 66 years, was admitted into the Louisville Marine Hospital on July 5th, laboring under phthisis pulmonalis at the third stage, affecting almost the entire left lung. In addition to this he had a copious, frequent and exhausting diarrhea. His skin was hot and dry, cheeks flushed, pulse very quick, tongue coated with a whitish fur; thirst urgent, appetite gone. Ordered six drops of the spts. pyroxylicus to be given three times a day. After having taken six doses the diarrhea ceased. The pulmonary disease, however, continued to progress, and in about three weeks terminated fatally. During the whole of this time the patient's bowels were perfectly regular.

*Autopsy.* Ulcers, varying in size from that of a dime to three quarters of an inch in diameter, were found at frequent intervals along the intestinal canal, from the pylorus to within an inch and a half of the anus. All of them occupied the entire thickness of the mucous coat, several involved also the muscular coat, and one or two had well nigh caused perforation of the intestine. Of the lesions, offered by the other parts and organs of the body, it is unnecessary to speak.

*Case* 2. *Diarrhea.*—Among the patients remaining in the hospital at the time I commenced my attendance there on the 4th of July last, was a Pennsylvanian named Martin, a thin, delicately formed man, 26 years of age, who was laboring under a diarrhea of several months' standing. He had taken almost the whole catalogue of astringents; had consumed large quantities of opiates, and had been twice salivated without producing even a temporary arrest of the disease. He was greatly emaciated, excessively feeble, so much so that he was scarcely able to get up to stool, without appetite and without hope of recovery. His pulse was small, feeble and slow; tongue covered in the center with a whitish fur, red at edges and tip. He had had ten, pale, watery dejections in the preceding twenty-four hours. No tenderness on pressure over abdomen, but a doughy feel, such as is said by authors to be characteristic of chronic peritonitis, but which, according to my experience, is observed also in other chronic bowel affections. Considerable *gargouillement* in right iliac fossa. Micturition attended by some burning; urine high colored and scanty. I prescribed a pill composed of tannin. gr. ij, morph. acet. gr. $\frac{1}{4}$, camphor gr. ij., to be taken every hour. Injection of tinct. opii and starch: Brandy toddy with a teaspoonful tinct. cinchon. c., occasionally during the day. Flaxseed poultice over abdomen. Hydrarg. cum cret. gr. x., plumb. acet. gr. iv., rhei grs. v., p. ipecac. gr. j., extr. hyoscyam. gr. v., to be made into pills of the requisite size and taken at bed time.

July 5th. Patient expressed himself as feeling no better. Number of discharges not less than on day before. His strength was quite as feeble and pulse no stronger; tongue, however, less coated. Prescriptions continued, increasing the quantity of tannin and substituting an injection of plumb. acet. for the one of tinct. opii, etc.

6th. Condititition unimproved. I now ordered nitrate of silver in small quantities internally in the form of pill and *per an.*, emplast. vesic. over abdomen.

7th. No change except the dark color imparted to the stools by the salt. Increase quantity of brandy, and the patient desiring a bowl of soup was allowed it. Continue prescriptions, adding p. ipecac. c. gr. x. on going to bed.

8th. Condition of the patient materially the same.—Had eight or ten discharges since last visit. Pulse small and very feeble; surface of body cool, extremities cold; countenance dejected, eyes sunken. He refused to take medicine any longer, urging that it did him more harm than good, and that he would die, in at most, a few hours. I now determined upon using the spts. pyroxylic., and prescribed it in doses of ten drops three times a day, which, after much persuasion, I prevailed on Mr. M. to take. All other prescriptions except the brandy were omitted.

9th. Patient had four discharges since previous morning. Thinks he is better. No perceptible alteration, however, in his general condition unless it be a slight increase in the force of his pulse. Continue prescriptions.

10th. Patient has had but one discharge. Tongue cleaned off. Extremities still cold, and surface of body generally below the usual temperature. Pulse same. Some appetite. Being averse to drinking any more brandy, which, he contends, has been injurious to him throughout, and desiring porter, the latter, with some scruples as to the propriety of the course, was allowed him in its stead.

11th. General expression of patient greatly improved. Has had but one dejection, which was in every respect natural. Porter and soup agreed well with him. The spts. pyrox. was ordered to be given only once during the day. Porter and soup continued, and in addition he was allowed two soft boiled eggs for dinner.

For two weeks the patient continued to improve, when, after too long exposure to the night air, the diarrhea returned with its former violence. The spts. pyroxylicus again cut it short, and the patient is now well. He after wards died of bronchitis. At the meroseopy the intestinal track was found entirely healthy.

*Case* 3. *Dysentery.*—Mr. A. F., a farmer, living two miles below Louisville, æt. 46 years, of vigorous constitution, though intemperate habits, was convalescent from a mild attack of cholera. On the morning of the 19th of July, after a restless and uncomfortable night, he was seized, while getting up, with pains in the back, loins, and abdomen, accompanied by a sense of general uneasiness, nausea and intense thirst. Soon after he had a painful and very small dejection of blood mixed with mucus. I saw him in three hours after this time. He had then had fifteen calls to stool. His skin was hot and dry, pulse somewhat accelerated and full; tongue coated with a white fur; thirst; anorexia; cephalalgia; vesical tenesmus; tenderness on pressure over whole course of colon. I prescribed spts. pyroxylicus gtt. x., to be taken in a wine-glassful of water every four hours. 6 P. M. Had but two discharges after first dose of medicine.

20th. 8, A. M. Slept well: no discharges since last visit. Felt so entirely restored that he had been to the field where the hands were digging potatoes, had brought some to the house, had them roasted, and with these and a couple of mutton chops had made a comfortable breakfast. Directing a dose of castor oil should it be necessary, I ceased my attendance.

*Case* 4. *Diarrhea.*—Sam, an able bodied negro, æt. 25, a blacksmith, belonging to C. Bradley, Esq., over worked himself at the forge on the afternoon of the 9th of August. That evening, about nine o'clock, after a sense of general uneasiness, he had a large frothy dejection, which left him very much exhausted. Thinking the advice of a physician unnecessary he took a dose of a nostrum known in this city as Forwood's cholera drops, and went to bed. He had five evacuations, similar in character to the one above mentioned, between this and morning. I saw him at daylight. He had just left the stool. His countenance was anxious, skin moist but warm, pulse full and quick; tongue covered with a whitish fur; thirst, nausea; pain on pressure over lower part of abdomen: mind flighty, from, I presume, the "drops," of which he had taken a considerable quantity. I prescribed 15 drops spts. pyroxylicus, to be taken every three hours: had a flaxseed poultice applied to abdomen, and directed brandy to be given every hour.

10 o'clock, A. M. Patient easy; one discharge after first dose of spts. pyroxilic. Continued prescriptions and added 10 grs. each of Dover's powder and submuriate to be administered at bedtime.

11th. Patient felt so well at the time he was to take the cal. and p. ipecac. c., that he omitted it. Since previous visit has had but one operation, which was almost natural.

Condition in every way satisfactory. Ceased my attendance.

*Case* 5. *Diarrhea.*—Mat. Horn, native of Ireland, æt. 21, laborer, admitted into the hospital on July 14th, was attacked on July 9th with pain in the stomach and bowels, followed by diarrhea. Has had from five to eight discharges daily since. *Condition at time of admission*—Skin natural; pulse full; anorexia; thirst; nausea; urine high colored and scanty; tenderness on pressure over epigastrium; *gargouillement* in right iliac fossa; tongue coated, clammy; dejections thin, copious and pale. Ordered spts. pyroxylic. gtt. xij., every four hours: flaxseed poultice to abdomen. Patient improved very rapidly for three days, when the medicine was discontinued. From exposure to the night air the disease recurred, but was again immediately checked by a few doses of the spts. pyroxylicus, and in the course of a short time the patient was discharged cured.

*Case* 6. *Diarrhea.*—Joseph Klean, native of Germany, æt. 38, a baker, admitted into the hospital on July 18th, laboring under diarrhea of seven weeks' standing. Had from twelve to fifteen yellow watery discharges in the twenty-four hours. Appetite good; surface of body natural; pulse slow and feeble; tongue covered with a yellowish fur; no tenderness over abdomen. Ordered, spts. pyroxylicus gtt. xij. every four hours. On the following morning the patient was better, and in a few days was able to assist in nursing his sick companions.

*Case* 7. *Dysentery.*—McCollom, marine, æt. 29, was admitted into the hospital on July 11th. Attacked with diarrhea two days before—has had from ten to twelve discharges during the twenty-four hours. Pulse slow and feeble, skin natural; tongue whitish. Ordered small doses of calomel, capsicum, camphor, opium, and brandy every hour.

12th and 13th. Marked improvement in condition of patient.

14th. Stools more frequent; condition same as on day of admission. Ordered, plumb. acet. gr. xx., pulv. opii, gr. v., and pulv. ipecac. gr. x. To be divided into ten powders, and one given every two hours; opiate injection after every discharge. At bedtime patient to take 15 gr. of Dover's powder.

15th. Patient slept well: Four discharges since last visit. Pulse and skin natural; tongue still furred; no appetite. Continue prescriptions.

16th. Passed a restless night; up nine times to stool; dejections small, thin, mixed with blood and accompanied by straining. The irritability of the rectum prevented the injections being retained. Tongue covered with a yellowish brown fur; pulse full and quick; considerable tenderness on pressure over abdomen; distressing tenesmus. Ordered cups over seat of greatest tenderness, followed by a flaxseed poultice, and, internally, fifteen grs. each of magnesia and sulph. sublimat. every three hours.

17th and 18th. Condition of patient materially the same. Ordered, spts. pyroxylic. gtt. xv., to be taken three times aday; poultices to be continued. In twelve hours after this time the disease had begun to ameliorate, and at the visit on the 19th, the patient expressed himself as being entirely relieved. In a few days after he was discharged cured.

*Case* 8. *Dysentery.*—Mr. Sullivan, laborer, æt. 30, entered the hospital on the 22d of July. Has been suffering for three months with dysentery; has from ten to twelve small, bloody stools during the day; has lost much flesh; appetite good; tongue coated with a white fur; skin natural; pulse slow; abdomen tender on pressure. At the suggestion of a medical friend in the city, I prescribed: ℞. Tannin., gr. xx., p. opii, gr. x., plumb. acet. gr. xxx., submur. hydrarg. gr. x.: M. To be divided into twenty-four powders, and one taken every two hours. Poultice to abdomen.

23d. Condition unaltered. Continue prescription.

24th. Six stools since visit. Ordered spts. pyroxylic. gtt. xij. three times a day.

25th. Has had three dejections; less tenesmus, less straining; tongue cleaning off. Complains of flatulence, for which I prescribed a pill composed of 1 gr. of ext. hyoscym. and gr. ss. of p. ipecac. to be taken every hour until relieved.

26th. Had six or seven stools. Increase the spts. pyroxylic. to xx. drops. The patient improved for a day or two under this treatment and then grew worse again. I persisted in the use of the spts. pyroxylicus until the 6th of August, when, finding that its influence over the disease was only temporary, I resorted to Hope's mixture, and under this and the hyoscyam. and ipecac, to relieve the flatulence, and Dover's powder at bedtime, the patient is improvıng. He is at present taking some of the chalybeate preparations, and gives promise of being soon entirely well.

*Case* 9, is one of dysentery, which has continued with unabated violence, notwithstanding every remedy which I have used. The spts. pyroxylicus, carried as high as thirty drops at a dose, and repeated three times a day, has been entirely ineffectual.

*Case* 10, except that it is of more recent date, is, in its main features and result of treatment, similar to case 9.

*Case* 11, is one of chronic diarrhea, in which the spts. pyroxylicus in large doses proved wholly inefficient, and which afterwards yielded in the course of a very few days to tannin, opium, calomel and acetate of lead.

*Case* 12. *Diarrhea.*—Syptoms, with the addition of tenderness over abdomen, similar to those of Joseph Klean. Treated successfully by the spts. pyroxylicus in doses of fifteen and twenty drops, and flaxseed poultices to abdomen.

*Case* 13. *Diarrhea.*—Jacob Ricker entered the hospital on the 9th of August, suffering from diarrhea and chronic rheumatism, affecting articulations of left side. Has between six and ten dejections during the twenty-four hours. Much emaciated, very feeble, no appetite; surface of body natural; pulse small, weak, 112; tongue furred; no tenderness on pressure over abdomen. The spts. pyroxylicus was given in doses of twelve drops, three times a day, for several days, without producing any appreciable effect on the bowel disease. The ordinary treatment was now adopted and continued for about an equal length of time, with like result. I determined to give the spts. pyroxylic. another trial, and, accordingly, ordered twenty drops to be taken every four hours. At the end of a few days the diarrhea was cured.

*Case* 14. *Dysentery.*—Odrad, an epileptic, for a long time an inmate of the hospital, was attacked about the middle of August with dysentery. The disease was accompanied by some febrile reaction. The tenesmus was almost constant. There was considerable tenderness, excited by pressure over the whole course of the colon, and also in other parts of the abdomen. Complete anorexia; distressing thirst. The spts. pyroxylicus, although administered for several days in doses of thirty drops, at intervals of four hours, proved entirely inefficient. I may remark that other remedies have since been used for a greater length of time without arresting the disease.

*Case* 15. *Diarrhea.*—Hoffman, after having labored under diarrhea and infiltration of the cellular tissue of left side for five or six weeks, was received into the hospital early in August. At time of admission his skin was pale; surface of body cool; pulse small and feeble; no tenderness over abdomen. Had five or six watery dejections during the day, and not less during the night. Anorexia; no thirst. Tongue pale and flabby. The spts pyroxylicus given at first in small and afterwards in larger quantities, produced no change in character nor diminution in number of dejections. Other treatment was substituted, under which patient improved.

*Case* 16. *Diarrhea.*—Armstrong, convalescent from a severe attack of parotitis, exposed himself to the sun, and was seized the following morning with diarrhea. I gave him twenty drops of the spts. pyroxylicus, in a few hours after taking which the discharges ceased. The next day he was admitted into the hospital complaining of excessive weakness and inability to work. He had no appetite; his skin was hot and tongue furred. I prescribed ol. ricin. ℥ j., which developed the diarrhea anew. Two doses of twenty drops each of the spts. pyroxylicus relieved him in a few hours.

*Case* 17. *Diarrhea.*—James Hughes, a robust Irishman, æt. 24, entered the hospital on the 13th of August. Seven days before he had a severe chill, followed by some febrile reaction. Copious fecal discharges, accompanied by griping, now supervened. The dejections soon became small, slimy and mixed with blood, and numbered from ten to twelve during the day. On day of admission his tongue and skin were natural; abdomen excessively tender; tongue heavily coated; had had nine dysenteric dejections in the preceding twenty-four hours.

Ordered hydrarg. chlorid. mit. gr. x, p. opii, gr. ij; flaxseed poultice to abdomen; laud. and starch enema after each discharge.

14th. Patient slept well; five stools since visit; tongue pale and flabby; pulse 72, full; tenderness of abdomen diminished. Twenty drops of spts. pyroxylic. were ordered to be taken three times a day. Continue poultice, Omit enema.

15th. One stool since visit; tongue, skin, and pulse natural; some appetite. Patient continued to improve and was on the eve of being discharged, when, during the night of the 20th, he was attacked with a very profuse diarrhea. Had had ten discharges when I saw him on the morning of the 21st. His skin was hot, pulse slightly accelerated; tongue furred; thirst; anorexia; no tenderness over abdomen. Prescribed spts. pyroxylic. gtt. 20, to be taken every three hours. Diet.

22d. Has had eight dejections. Condition same. Continue medicine.

23d. Has had one stool, which was in all respects natural.

24th. Discharged.

*Case* 18. *Diarrhea.*—J. Mooney, æt. 21, admitted on 21st Aug. Had had diarrhea for four days. Twenty thin, pale dejections in last twenty-four hours. No appetite; tongue covered with a whitish fur; pulse and skin natural; *gargouillement* in both iliac fossa; some tenderness over abdomen. Ordered spts. pyroxilic. gtt. xx every four hours; diet.

22d. Had seven dejections last night. None during day. Continued medicine.

23d. Had no calls to stool till night, when he was up three times.

24th. Convalescent.

*Case* 19. *Dysentery.*—After having finished my visit at the hospital on the morning of the 20th of August, I was desired to return to see Mr. Morin, who had just been admitted for what was said to be cholera. He had been found a few hours before in one of the streets near the river, so much exhausted that he was unable to stand up, and purging every few minutes. I learned from him that about three weeks ago he had a severe attack of diarrhea, from which he partially recovered. Six days ago the disease recommenced, accompanied by cramps in the stomach, nausea, headache, and pains in the back. The dejections were at first unattended by pain, were loose, pale and copious. Gradually, however, griping came on, which soon became so distressing that he could hardly leave the stool, and the discharges were small, bloody and excessively offensive.

When I saw him he was so weak as scarcely to be able to turn over in bed. His skin was hot and dry; pulse 108, full and hard; tongue red at edges and tip, coated in center; urine high colored and scanty; has had innumerable stools in last twenty-four hours, attended with much pain, and bloody; abdomen painful on pressure. Ordered cups over seat of pain, followed by a warm poultice; laud. and starch injection after each discharge; spts. pyroxylic. gtt. xx every four hours.

21st. Patient says he has been to stool every fifteen minutes, though only twice was the call accompanied by pain, or the discharges bloody. Surface of body warm, bathed in perspiration; tongue covered with a white fur; pulse 122, but soft; tenderness over abdomen continues. Continue poultices, injections, and spts. pyroxylic.

22d. Has had ten dejections, only one of which was painful. Tongue still coated; skin and pulse natural; no tenderness over abdomen. Omit poultices, continue injections, and give of spts. pyrox. in ten drop doses every hour.

23d. Had eight discharges since visit—a few of them were attended by griping. Skin and pulse same; tongue still coated, though less so than yesterday; thirst; no appetite. Continue prescriptions.

24th. Had some discharges; no griping; skin and pulse same; tongue cleaned off; some appetite; thirst still continues. Ordered spts. pyroxylic, gtt. x every hour.

25th. Number and character of discharges same; rested badly; complains of exhaustion; no appetite; tongue again coated; abdomen presents doughy feel noticed in another case. The spts. pyroxylic. was now omitted; pil. hydrarg. in combination with opium was administered; in the course of the next twenty-four hours there was a marked amelioration in the condition of the patient. The disease, however, still continued, and in a few days was not less alarming than before. The diluted sulphuric and nitric acid were substituted for the blue mass and opium, and the patient is at present convalescent.

*Case* 20. *Diarrhea.*—A German, who entered the hospital to be treated for phthisis, was attacked some days after with very profuse diarrhea, accompanied by well marked febrile disturbance. His face was flushed, skin hot, mouth dry; no tenderness on pressure over abdomen. Dejections thin, copious and frequent. Prescribed spts. pyroxylic. gtt. xx every three hours. In twenty-four hours after the diarrhea had ceased entirely.

*Case* 21. *Diarrhea.*—A hospital patient, convalescent, from intermittent fever, was seized with diarrhea. The dejections amounted to as many as ten or a dozen in the twenty-four hours. The spts. pyroxylic. administered in same doses as above checked the disease in less than twelve hours.

*Case* 22. *Diarrhea.*—Mr. Patterson, an Englishman, under treatment in the hospital for salivation, produced by greasing his pants with mercurial ointment in order to rid himself of vermin, with which he had been much annoyed, was attacked with diarrhea. The disease created no febrile reaction, was unaccompanied by tenderness of the abdomen, and did not affect his appetile. The spts. pyroxylic. in twenty drop doses given every three hours greatly mitigated, without, however, entirely arresting the discharges. In a couple of days the disease recommenced, and continned, notwithstanding the exhibition of spts. pyroxylic. in large quantities at frequent intervals, until I finally changed the treatment, soon after which the patient grew better.

*Case* 23. *Diarrhea.*—Late in the evening of one of the last days of July, I was called to the country. The nights were unusually cool for the season. While returning home I became chilled, and a short time afterwards went to bed with a slight fever. I rested badly, and awoke in the morning with a diarrhea. The first discharge was copious and watery, and left me much exhausted. I immediately swallowed fifteen drops of the spts. pyroxylic. in a glass of brandy and water. The indications for the use of stimuli being present, I took small quantities of brandy at short intervals. At 12 o'clock, M., I had another discharge similar in character to the first. I repeated the spts. pyroxylic. There being some tenderness over abdomen, my friend Dr. Breckinridge, suggested the use of dry cups. They were applied and afforded much relief. Having no appetite, much thirst and slight febrile disturbance, I took at bed time fifteen grs. of pil. hydrarg., eight grs. of ext. hyoscyam., and one gr. of ipecac. After the operation of the mercury on the following morning, I felt no more uneasiness of bowels or tenderness over abdomen, and that evening was sufficiently restored to visit my patients.

The dose of the spts. pyroxylic., as stated by Christison, is five minims, and "is conveniently given with a drachm of compound tincture of cardamom made into an ounce mixture with water merely." I have found this combination unnecessary, its taste when diluted with water being comparatively but slightly unpleasant.

I am of the opinion that five minims is much too small a dose, at least when given in diarrhea or dysentery. It will be remarked that I have varied the dose of the medicine in different cases. I commenced by prescribing it in the quantities used by Dr. Lavirotte, and in some instances with the happiest effect. In others again, however, I was obliged to increase the amount before any good results were manifest. It appears to me from my brief experience with the remedy, that it is most frequently efficacious when given in doses of twenty drops at intervals of three hours.

It will be remembered that the pyroxylic spirit, under the incorrect designation of *naphtha*, was highly extolled a few years ago by Dr. Hastings, as a remedy for pulmonary consumption; "and has since come into general use as a paliative for that disease, and also as a powerful anti-emetic drug in cases of obstinate vomiting."

According to the Editor of the Pharmaceutic Journal, the naphtha of Dr. Hastings is not pyroxylic spirit, but pyro-acetic spirit, acetone, or hydrated oxide of mesityle of Kane, ($C^6$ $H^5$ O + aq.) But all the so-called medicinal naphtha of the Edinburg shops, obtained from London, is pyroxylic spirit." (Christison.) This is true also, I imagine, of the naphtha sold in the drug stores of our own country.

It is prepared, according to Dr. Ure, by uniting the crude pyroligneous acid with lime, and then distilling the pyrolignite of lime, which yields about one per cent. of crude spirit. The spirit is purified by repeated distillation from quick lime. It is then a pale straw-yellow, mobile fluid, of a powerful penetrating odor of wood smoke. (Christison.)

In the Medical Examiner, for August, I notice a communication by Dr. Henry T. Child, of Philadelphia, on the use of this remedy in cholera. He details four cases, the following abstract of which may not be uninteresting:

Case 1st, had had diarrhea for five days. Opium, kino and camphor arrested the disease for a few hours. At the expiration of this time, patient was attacked with vomiting of "rice water" and copious watery diarrhea. His pulse was small and corded; eyes sunken; countenance and hands shrivelled. Fifteen drops of the spts. pyroxylicus were taken; the vomiting, which had been incessant, cased immediately, and did not return. The cure was completed by the exhibition of mercury.

Case 2d. had labored under diarrhea for six hours.

The discharges assumed the characteristic "rice water" appearance, and there was vomiting of a similar fluid. Ten grains of calomel and fifteen drops of the spts. pyroxylicus were given every hour—one dose each half hour. Vomiting immediately checked and did not return; in three hours the dejections became bilious. Convalescence rapid.

Case 3d, had had diarrhea for two days. Was in a profuse cold perspiration; no pulsation in radial artery; briefly, all the symptoms of fatal collapse. A *teaspoonful* of spts. pyroxylicus was given; to be followed in fifteen minutes by submur. hydrarg. gr. xv.; the dose to be repeated every fifteen minutes. Convalescent next day.

Case 4th, was attacked suddenly with watery diarrhea and nausea. Spts. pyroxylic. was prescribed in doses of ten drops, hydr. submur. in five gr. doses every hour.

It will be observed that in the above cases the spts. pyroxylic. was combined with calomel. In those that I have reported, the remedy was tried single handed, though followed, it is true, whenever indicated by medicines calculated to correct the disordered secretions.

Touching the *modus operandi* of the spts. pyroxylicus, I confess myself totally at a loss to determine. I can but ask, with Dr. Lavirotte—Does this medicine act like opium and the other calmants, as alum and colombo, and the other astringents, as nitrate of silver, by substitution, or rather has it a special action which modifies the intestinal secretion? It is too easily borne to warrant its being compared to nitrate of silver; and its antispasmodic properties are, to say the least, very insignifiicant; while its nanseous, sharp and bitter savor would seem to indicate that it exercised an astringent influence.

On this subject Christison speaks as follows: "Its actions have not yet been carefully investigated; but it seems to be narcotic, sedative, and calmant. It certainly possesses the property of allaying the cough and febrile excitement of phthisis pulmonalis; but general experience has not borne out the sanguine hopes entertained by Dr. Hastings of its efficacy as a remedy in that disease. I can amply confirm all that has been said of it as an anti-emetic remedy in cases of chronic vomiting; for in cases of this affection, depending on both functional and organic diseases, I have frequently seen the vomiting arrested or greatly mitigated by pyroxylic spirit. Even creosote has appeared to be scarcely as efficacious."

It will be oberved, however, that he does not allude to its use in the treatment of diarrhea.

The object of this paper has been to call the attention of the profession to this agent in the treatment of two of the most common of bowel diseases, in order that its virtues might be fully tested. I am well aware that the first requisite for a successful comparison of therapeutic or other results, is a sufficient similarity between the number of collated cases, and in addition to this, to use the language of a distinguished Parisian physician, "the data must be numerous, collected at different times, in different places, and, if possible, by several observers." For "it is easily seen," he continues, "that the results of a number of facts too limited, collected in a short space of time, in a single place, and by a single observer, however exact as regards that series of data, may yet be very different from, or even the reverse of conclusions drawn from a larger series and one collected under various circumstances."

I have prescribed the spts. pyroxylicus with marked success in several cases of bowel disease occurring in children.

I am at this time treating both in the hospital and in private practice, a number of cases of diarrhea and dysentery, with this agent. My success in these and in some other cases that I have omitted in the present communication, I purpose publishing in some future number of this Journal.

